# Impact of systematic screening for social determinants of health in a level IV neonatal intensive care unit

**DOI:** 10.21203/rs.3.rs-4656439/v1

**Published:** 2024-07-26

**Authors:** Joanne Lagatta, Caitlin Hoffman, Melissa Harris, Krishna Acharya, Margaret Malnory, Susan Cohen

**Affiliations:** Medical College of Wisconsin; Medical College of Wisconsin; Medical College of Wisconsin; Medical College of Wisconsin; Medical College of Wisconsin

**Keywords:** social determinants of health, prematurity, neonatal intensive care

## Abstract

**Objective:**

To determine whether screening for social determinants of health (SDoH) in a level IV neonatal intensive care unit (NICU) could uncover additional family needs.

**Methods:**

Secondary analysis of a prospective study in a level IV NICU. Participants filled out the Protocol for Responding to and Assessing Patients’ Assets, Risks and Experiences (PRAPARE) tool, which includes economic, housing, transportation, and safety questions. Questionnaires were completed via secure tablet; the research team notified social workers of reported needs. Illness and demographic characteristics were compared between families who did and did not report resource needs. Manual chart review assessed subsequent response to reported SDoH needs.

**Results:**

Of 319 respondents, 61(19%) reported resource needs. Of 61 families, 88% received repeat social work encounter to re-assess for resources; 59% received new resource referrals.

**Conclusions:**

Systematic SDoH screening can identify needs throughout the NICU stay, even among families already connected to social work support.

## Background

Social determinants of health (SDoH) are associated with preterm birth and its complications. SDoH, as defined by the World Health Organization, encompass a variety of non-medical factors that affect a person’s health and include education, income, basic needs such as access to healthy foods and safe housing, and social support networks.^1–3^ For infants requiring care in a neonatal intensive care unit (NICU), SDoH have been found to increase risk of prematurity, low birth weight, and complications related to prematurity.^4–14^ For parents of infants in the NICU, SDoH are associated with less ability to spend time in the NICU as well as higher rates of maternal mental health diagnoses.^15,16^ A recent statement by the American Academy of Pediatrics endorsed the need to address SDoH in the NICU.^17^ as a strategy to improve the quality of healthcare delivered in this high-risk patient population.

Systematic screening tools to identify SDoH needs have been developed for use in healthcare settings, aiming to allow healthcare staff and providers to identify a broader array of needs than would typically be covered in a usual clinic or hospital visit. Using these tools in the inpatient setting has advantages and disadvantages. In a study conducted in an emergency department, staff reported concerns with time constraints, while patients reported concerns with vulnerability given rapid turnover of the emergency room.^18^ In the NICU, where patients often experience a prolonged stay, staff may have more opportunities to establish rapport with families; thus the NICU setting may be ideally poised to conduct such screenings. In one NICU that conducted SDoH screening, over 80 percent of families screened had at least one unmet need, and 98% of those screens resulted in referrals to meet those needs.^19^ Nonetheless, only one in four NICUs currently uses a screening tool to identify SDoH related needs.^20^ While healthcare professionals including those in NICU settings have been found to be in support of universal SDoH screening, many also share hesitation related to implementation barriers, current level of knowledge with screening, lack of resources to respond to uncovered needs, barriers in translating resources from inpatient to outpatient settings, and preference to establish individual rapport rather than relying on screening tools.^21–25^ In a setting like the NICU which may have more resources or existing processes to screen and refer to needed resources, it is unknown how a systematic screening protocol would integrate into existing supports.

The objective of this study was to determine whether systematic screening for SDoH in a single NICU, in addition to existing social work and psychologist support, would uncover additional social needs for families, and whether screening could facilitate addressing any newly identified needs.

## Methods

This was a secondary analysis of a prospective study conducted from September 2018 – March 2020, which received approval from the Children’s Wisconsin Institutional Review Board.^25,26^ It was a single-center study conducted in a level IV 70-single-bed NICU with a fetal consult coordinating center and connected to a delivery hospital. The NICU does not designate any beds as lower or higher acuity. Infants and families had access to 3 social workers, a dedicated psychologist for NICU families, case management, and a March of Dimes family support coordinator. Infant-parent dyads were eligible for the prospective study if the infant had a length of stay of at least 14 days and was anticipating discharge home with their parent.

Parents who participated in the study were asked to fill out a demographic questionnaire as well as questions from the Protocol for Responding to and Assessing Patients’ Assets, Risks and Experiences (PRAPARE) tool. The PRAPARE questionnaire includes national core measures and community priorities, including race and ethnicity, resource needs (food, utilities, clothing, transportation, and housing status), safety (neighborhood and domestic violence), mental health (including presence of emotional distress disorder and stress level), social support (number of adults living at home, amount of weekly social interaction, and number of adults nearby to help care for the infant), education level, employment and household income level, insurance status, and language preference.^27^ These questionnaires were completed at the bedside on a digital tablet. For any identified concrete needs, which included worry about housing, financial limitations, lack of transportation, or safety, the research coordinator immediately notified the social work team of that need. *Housing need* was defined as answering “yes” to the question “Are you worried about losing your housing?” or answering, “I don’t have housing” in response to the question “What is your housing situation?”. *Safety need* was defined as answering “no” to the question “Do you feel physically and emotionally safe where you currently live?” or answering yes to the question “In the past year, have you been afraid of your partner or ex-partner?” *Transportation need* was defined as answering “yes” to the question “Has a lack of transportation kept you from medical appointments, meetings, work, or from getting things needed for daily living?” *Financial need* was defined as answering “yes” to the question “In the past year, have you or any family members you live with been unable to get any of the following when it was really needed?” with options including food, utilities, clothing, childcare, medicine or health care, phone or other.

Infant illness variables were collected by manual chart review. These included day of life at interview, gestational age, birth weight, congenital and chromosomal anomalies, days on a ventilator, vasopressor administration, days of life at discharge, corrected gestational age at discharge, respiratory support at discharge, feeding support at discharge, total number of medications at discharge. Post-discharge variables included readmissions to the hospital and emergency department visits within 3 months of discharge, consistent with previously collected study data. Demographic variables were collected by parent report at enrollment and included parent age, race/ethnicity, insurance type, education level, transportation method (own car, rides from friend or family, public transportation), distance from home to hospital, number of adults living at home, income level, and childcare plan (parent or family, daycare or nanny, or unknown plan).

After discharge, a manual chart review of the electronic health record assessed what had been done to address needs identified by parents on the PRAPARE questionnaire, which included concerns regarding housing, finances, transportation, or safety. Social work and nursing documentation were reviewed manually to determine whether new referrals were placed, or resources were provided to address the identified needs. Documentation was categorized into three categories. *Screened and referred* was defined as documentation that the social work team had screened a family in response to identified needs on or after the date of the PRAPARE questionnaire, and referred to new resources, with no prior notes that already addressed that specific need. *Screened* was defined as documentation that the social work team had screened a family in response to the identified need, but did not refer to new resources above what had already been provided prior to the date of the study questionnaire. *No screen* was defined when no documentation of encounter followed the PRAPARE questionnaire.

It was expected that for resource needs including transportation, housing, and financial concerns there would be explicit social work documentation of how the specific need was addressed. Based on discussion with the social work team it was understood that responses to safety concerns would be confidential and are typically not documented explicitly in the patient chart. For these concerns we relied on notes taken by the research coordinator about their follow-up with the social work team during the study period and social work notes documented over the course of the hospitalization.

### Statistical Analysis

Infant illness characteristics and parent demographics were compared between those families who reported a resource need and those families who did not report resource needs. Chi-squared or Fisher’s exact tests were used to compare differences in proportions; Kruskal-Wallis tests were used to compare differences in medians. For families who reported a resource need, we used descriptive statistics to illustrate the details of what types of resources were provided, or any context we were able to determine for families who did not receive new resources. STATA version 16.1 (College Station, TX) was used for analyses.

## Results

A total of 319 parents enrolled in the study, and 19% (61/319) reported at least one resource need. Tables 1 and 2 show infant illness and parent demographic characteristics of those who did versus did not identify resource needs on the PRAPARE questionnaire. Infants whose parents reported resource needs were more likely to be born extremely preterm, to require surgery and vasopressors in the NICU, and to experience a longer NICU length of stay. After discharge, they were more likely to be seen in the emergency room. Parents who reported resource needs were more likely to report Black race and/or Hispanic ethnicity, less education, public insurance, lower income and less social support.

**Figure 1:**
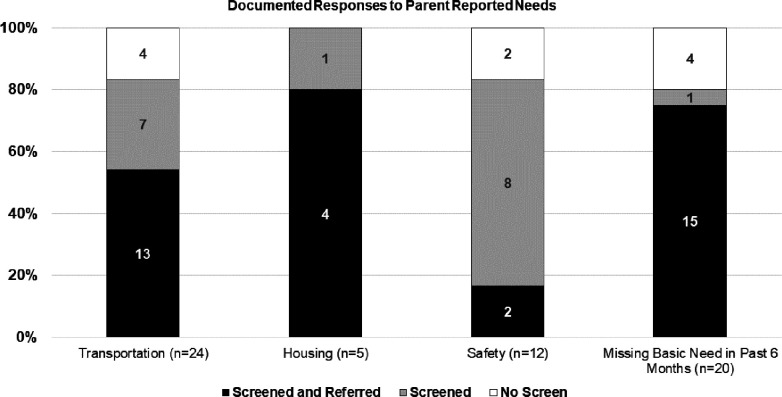
Documented responses to parent reported needs on screening. Figure 1 shows counts of responses to parents’ report of SDoH needs on the Protocol for Responding to and Assessing Patients’ Assets, Risks and Experiences (PRAPARE) tool. *Screened and referred* was defined as documentation that the social work team had screened a family in response to identified needs on or after the date of the PRAPARE questionnaire, and referred to new resources, with no prior notes that already addressed that specific need. *Screened* was defined as documentation that the social work team had screened a family in response to the identified need, but did not refer to new resources above what had already been provided prior to the date of the study questionnaire. *No screen* was defined when no documentation of encounter followed the PRAPARE questionnaire.

Of the 61 parents who identified needs, 24 identified transportation needs, 5 identified housing needs, 12 identified safety needs, and 20 identified other economic needs (Fig. 1). Most parents identified only one need (51/61, 84%), with 10 identifying multiple needs. Figure 1 shows the documented response by type of identified need. Additional resource referrals were documented for 56% of positive transportation screens, 80% of positive housing screens, 20% of positive safety screens, and 79% of positive economic screens. Of the 61 families who identified needs, 36 (59%) resulted in documentation of a new resource referral.

Table 3 documents details of the responses to identified needs on screening. For those with transportation needs, most families were referred to a medical transportation company for assistance; one family was given resources on local bus routes. For families with housing needs, most were referred to the Ronald McDonald House and one family was given transportation resources. Those with economic needs received a mix of resources; some received Ronald McDonald House or medical transportation referrals and others were given resources for food, finances, employment, and housing. For the 12 safety concerns, several families were already in close contact with social work at the time of the screening, and all but 2 families who were not already in contact with social work were screened immediately after the survey. Table 3 also shows details of documented responses to families whose screen indicated a need who received a social work screening encounter but no new referrals. Most of these concerns were related to transportation to the NICU itself, and the families had arranged transportation by the time of the encounter. Housing questions that did not result in new referrals were often issues related to delays in getting temporary housing close to the hospital. Some safety referrals had documentation that the issue was related to an ex-partner or a former housing situation that was no longer a concern.

There were 14 families for whom additional screening by social work was not noted after families reported a need via PRAPARE screening. Of these families, 6 had no social work documentation and were discharged within 72 hours of the screen; another 6 had previous documentation from social work about referrals to resources. For 2 families, research coordinator notes confirm that social work was notified, but we were not able to find documentation of subsequent conversation in the chart or research team notes.

## Discussion

We took advantage of an opportunity embedded in a prospective study of parent experiences related to infant health in and after NICU discharge to understand the potential impact of systematic SDoH screening in a level IV NICU. More than half of needs reported on study screening resulted in a new resource referral, even in a well-resourced NICU with social work, case management, and psychology support. Some positive screens ultimately identified issues that were no longer relevant by the time of encounter, which offers insight into design considerations for SDoH screening in inpatient settings like the NICU.

SDoH influence a wide range of health, individual and family level functioning;^27^ inequities in SDoH are one of the root causes of health disparities.^28^ SDoH are known to be associated with a higher risk of preterm birth, and infants born preterm or with chronic conditions are at higher risk for morbidity and mortality in the NICU. Unaddressed SDoH needs can place vulnerable populations at even higher risk. Consistent with these data, we found that infants whose parents identified resource needs on screening were more likely to be born extremely preterm, to require more intensive care unit interventions, and to have a longer NICU stay. Our findings endorse that screening for SDoH in the NICU presents an opportunity to better address the needs of our highest-risk patients and families. It is also important to note the context for our finding that 19% of families identified resource needs. In this study cohort, almost half were privately insured, most owned cars, lived in a 2-parent household, and had some college education. Our NICU has multiple staff members dedicated to providing family support already. The proportion of families identifying resource needs may be higher in NICUs serving a different patient population or with fewer staff resources already in place.

We found that more than half of positive screens led to additional resource referrals, even with existing family support processes in place. The NICU in some ways presents an ideal opportunity for health care teams to understand and address the needs of their patients and families over time, due to the length of time often required before discharge home. Nurses and social workers are trained to establish rapport and build trust and identify ways to support families through those relationships. Unfortunately, families with fewer financial resources, other children at home, less ability to take time off work, or other competing issues may have less ability to be present at the bedside to form those relationships. We also found that some families’ circumstances changed over the course of their child’s stay in the NICU and they found themselves needing resources that were not needed earlier during the admission. Using an electronic tablet-based method of screening in this study was advantageous as it allowed parents to fill out a survey when they were available and then social workers were promptly notified of resource needs so that they could address these needs with families.

We also found that many positive screens did not result in additional resource referrals. One issue with adapting a questionnaire such as PRAPARE to an inpatient setting is that a family’s circumstances may be very different in their daily life compared to what they face in the NICU. In the NICU or other inpatient settings, it would likely be beneficial to specify whether needs are temporary and related to the hospital stay or whether they are more long-term needs, as depending on the temporality of the need, different resources may be provided. This was especially evident when it came to screening for housing and transportation needs; some families required temporary housing closer to the hospital as they lived too far away to reasonably commute and some also required transportation to and from the hospital during the NICU stay. Other families indicated the same needs, but they were more long-term as they were experiencing housing instability or did not own a personal vehicle for transportation. Our screening approach was embedded within an ongoing research study and not administered by clinical staff; therefore, some screens identified resource needs that had already been addressed, and others were identified close to discharge such that repeat encounters may have been less feasible. Implementation of systematic screening approaches in the real world should leverage the benefit of clinical context. This could also include establishing a method to communicate SDoH screening information to outpatient partners, as we observed that some families who were receiving transportation resources in the NICU went on to miss outpatient follow-up appointments due to transportation needs that were not continued after discharge. In children’s hospital NICUs, 63% of neonatal follow-up clinics now screen for SDoH in the outpatient setting.^31^ Since the completion of this study, our neonatal follow-up clinic team has adapted a process to assess for SDoH needs prior to discharge in order to identify resources that could help improve the transition home.

Strengths of this study include the opportunity to test a systematic screen for SDoH in addition to existing family support services, manual chart review of responses, and the broad clinical and demographic representation in this study cohort. There are several limitations. We do not know whether some responses were already in process by the time of the screen, or whether some responses occurred but were not documented, as might especially be expected with safety concerns. We only obtained parent report from those who agreed to participate in the original study; although we enrolled 84% of eligible infant-parent dyads, we do not have parent-reported resource need information from parents who did not participate.

The results of this study highlight that systematic screening for SDoH in a NICU has the potential to identify needs throughout the NICU stay, even among those who are already connected to social work and have previously received support. Inpatient settings with a long stay and especially with families far from home may require more consideration of adapting settings to reflect both acute needs related to hospitalization and longer-term resource needs.

## Figures and Tables

**Table 1:** Infant clinical characteristics of families screening for resource needs.

Variable	Category	All	Indicated need?		p
			No	Yes	
NICU ILLNESS					
**Day of life on date of questionnaire, median (IQR)**		21 (17–30)	19.5 (16–28)	27 (20–37)	**<0.001**
**Gestational age**	<=28 weeks	71 (22%)	49 (19%)	22 (36%)	**0.016**
	29–33 weeks	113 (35%)	95 (37%)	18 (30%)	
	37 + weeks	135 (42%)	114 (44%)	21 (34%)	
**Birth weight in kilograms, median (IQR)**		1820 (1130–2624)	1930 (1220–2700)	1560 (860–2270)	**0.007**
**Surgery**	No	180 (56%)	153 (59%)	27 (44%)	**0.033**
	Yes	139 (44%)	105 (41%)	34 (56%)	
**Postnatal steroids**	No	248 (78%)	205 (79%)	43 (70%)	0.130
	Yes	71 (22%)	53 (21%)	18 (30%)	
**Ventilator days**	0 days	140 (44%)	118 (46%)	22 (36%)	0.088
	1–7 days	90 (28%)	76 (29%)	14 (23%)	
	8–30 days	38 (12%)	28 (11%)	10 (16%)	
	> 30 days	51 (16%)	36 (14%)	15 (25%)	
**Vasopressors used**	No	246 (77%)	206 (80%)	40 (66%)	**0.017**
	Yes	73 (23%)	52 (20%)	21 (34%)	
**Number of consultants involved**	<=1 consultant	107 (34%)	92 (36%)	15 (25%)	0.100
	2 + consultants	212 (66%)	166 (64%)	46 (75%)	
**Days of life at discharge, median (IQR)**		42 (28–85)	40 (26–77)	57 (35–102)	**0.005**
**Respiratory support at discharge**	Room air	221 (69%)	182 (71%)	39 (64%)	0.102
	Home oxygen	76 (24%)	62 (24%)	14 (23%)	
	Tracheostomy	22 (7%)	14 (5%)	8 (13%)	
**Feeding support at discharge**	All oral	219 (69%)	175 (68%)	44 (72%)	0.088
	Nasogastric tube	35 (11%)	33 (13%)	2 (3%)	
	Gastrostomy tube	65 (20%)	50 (19%)	15 (25%)	
**Total number of medications at discharge**	0 medications	159 (50%)	134 (52%)	25 (41%)	0.066
	1–2 medications	86 (27%)	71 (28%)	15 (25%)	
	3 + medications	74 (23%)	53 (21%)	21 (34%)	
**Number of appointments scheduled post-discharge**	<=1	68 (21%)	57 (22%)	11 (18%)	0.142
	2–3	109 (34%)	93 (36%)	16 (26%)	
	4+	142 (45%)	108 (42%)	34 (56%)	
**POST-NICU ACUTE HEALTHCARE UTILIZATION**					
**Number of readmissions**	0 readmissions	236 (74%)	186 (72%)	50 (82%)	0.104
	1 readmission	62 (19%)	56 (22%)	6 (10%)	
	2 + readmissions	21 (7%)	16 (6%)	5 (8%)	
**Number of emergency department visits**	0 visits	221 (69%)	187 (72%)	34 (56%)	**0.007**
	1 visit	64 (20%)	43 (17%)	21 (34%)	
	2 + visits	34 (11%)	28 (11%)	6 (10%)	

Infant clinical characteristics of families who indicated a resource need based on screening, defined as a positive response to transportation, housing, economic, or safety concerns on the Protocol for Responding to and Assessing Patients’ Assets, Risks, and Experiences (PRAPARE) tool. Included characteristics on the table were different between parents who did and did not indicate needs on screening with a p value < 0.2; bold p values highlight those with p values < 0.05.

**Table 2:** Parent demographic characteristics of families screening for resource needs.

Variable	Category	All	Indicated need?		P
			No	Yes	
**DEMOGRAPHICS**					
**Parent age, median (IQR)**		30 (26–34)	31 (26–34)	27 (23–34)	**0.023**
**Race/ethnicity**	Black	71 (22%)	47 (18%)	24 (39%)	**< 0.001**
	White	202 (63%)	183 (71%)	10 (31%)	
	Asian	8 (3%)	5 (2%)	3 (5%)	
	Other	4 (1%)	3 (1%)	1 (2%)	
	Hispanic	34 (11%)	20 (8%)	14 (23%)	
**Insurance type**	Public	174 (55%)	122 (47%)	52 (85%)	**< 0.001**
	Private	145 (45%)	136 (53%)	9 (15%)	
**Education level**	< High school	22 (7%)	8 (3%)	14 (23%)	**< 0.001**
	High school	62 (19%)	46 (18%)	16 (26%)	
	Some college	235 (74%)	204 (79%)	31 (51%)	
**Transportation method**	Own car	259 (81%)	226 (88%)	33 (54%)	**< 0.001**
	Rides from friend	48 (15%)	27 (10%)	21 (34%)	
	Public transportation	12 (4%)	5 (2%)	7 (11%)	
**Number of adults who live at home**	Single parent	32 (10%)	22 (9%)	10 (16%)	0.142
	Parent +1	261 (82%)	216 (84%)	45 (74%)	
	> 2 adults	26 (8%)	20 (8%)	6 (10%)	
**Number of adults who live nearby**	No adults nearby	38 (12%)	31 (12%)	7 (11%)	0.171
	1–2 adults	99 (31%)	74 (29%)	99 (31%)	
	> 2 adults	181 (57%)	152 (52%)	181 (57%)	
**Income level**	<$20,000	64 (20%)	38 (15%)	26 (43%)	**< 0.001**
	$20,000-$40,000	60 (19%)	46 (18%)	14 (23%)	
	>$40,000	162 (51%)	151 (59%)	11 (18%)	
	Prefer not to answer	33 (10%)	23 (9%)	10 (16%)	
**Childcare**	Parent or relative	248 (78%)	196 (76%)	52 (85%)	**0.030**
	Daycare or nanny	64 (20%)	58 (22%)	6 (10%)	
	Unknown	7 (2%)	4 (2%)	3 (5%)	
**How often do you see or talk to people that you care about and feel close to?**	<=2x week	49 (16%)	34 (13%)	15 (25%)	**0.020**
	>=3x week	267 (85%)	223 (87%)	44 (75%)	
**Stress is when someone feels tense, nervous, anxious, or can’t**	Not at all-moderate	237 (75%)	197 (77%)	40 (68%)	0.157
**sleep at night because their mind is troubled. How stressed are you?**	Quite a bit-very much	79 (25%)	60 (23%)	19 (32%)	

Self-reported characteristics of families who indicated a resource need based on screening, defined as a positive response to transportation, housing, economic, or safety concerns on the Protocol for Responding to and Assessing Patients’ Assets, Risks, and Experiences (PRAPARE) tool. Questions about social and emotional health are drawn from the PRAPARE tool and are included verbatim. Included characteristics on the table were different between parents who did and did not indicate needs on screening with a p value < 0.2; bold p values highlight those with p values < 0.05.

**Table 3:** Response details after screening.

Screen and subsequent referral	
Transportation need referrals (n = 13)	Medical transportation company referral for rides, bus tickets or mileage reimbursement (n = 13)
	Review of bus routes with social worker (n = 1)
Housing need referrals (n = 4)	Ronald McDonald House (n = 4)
	Medical transportation company referrals (n = 1)
Economic need referrals (n = 13 which were not duplicated from transportation and housing)	Food, financial, employment, and/or housing resources provided (n = 6)
	Medical transportation company referrals (n = 5)
	Ronald McDonald House (n = 4)
**Screen but no referral**	
Transportation	Arranged alternative transportation in the interim
Housing	Most notes indicate that parents lived locally and had vehicle
Safety	Majority denied safety concerns; one safety response was already in process
Economic	Had already received resources at referring hospital

Details of documented encounters for parents who indicated a resource need based on screening, defined as a positive response to transportation, housing, economic or safety concerns on the Protocol for Responding to and Assessing Patients’ Assets, Risks, and Experiences (PRAPARE) tool. Notes reflect manual chart review of nursing, case management and social work encounters documented after the date of positive screening.

## Data Availability

The datasets generated and analyzed are available from the corresponding author on reasonable request.

## Abbreviations

NICU: neonatal intensive care unit
SDoH: social determinants of health
PRAPARE: Protocol for Responding to and Assessing Patients’ Assets, Risks and Experiences
